# A Low-Cost, Portable, High-Throughput Wireless Sensor System for Phonocardiography Applications

**DOI:** 10.3390/s120810851

**Published:** 2012-08-07

**Authors:** Akkarapol Sa-ngasoongsong, Jakkrit Kunthong, Venkatesh Sarangan, Xinwei Cai, Satish T. S. Bukkapatnam

**Affiliations:** 1 School of Industrial Engineering & Management, Oklahoma State University, Stillwater, OK 74078, USA; E-Mail: akkarap@okstate.edu; 2 Boonjitwitthaya School, Sriracha, Chonburi 20230, Thailand; E-Mail: jakkrit.kunthong@gmail.com; 3 Tata Consultancy Services, Chennai 600042, India; E-Mail: venkatesh.sarangan@tcs.com; 4 Department of Computer Science, Oklahoma State University, Stillwater, OK 74078, USA; E-Mail: xinwei@cs.okstate.edu

**Keywords:** heart sound, phonocardiography applications, wireless sensor system

## Abstract

This paper presents the design and testing of a wireless sensor system developed using a Microchip PICDEM developer kit to acquire and monitor human heart sounds for phonocardiography applications. This system can serve as a cost-effective option to the recent developments in wireless phonocardiography sensors that have primarily focused on Bluetooth technology. This wireless sensor system has been designed and developed in-house using off-the-shelf components and open source software for remote and mobile applications. The small form factor (3.75 cm × 5 cm × 1 cm), high throughput (6,000 Hz data streaming rate), and low cost ($13 per unit for a 1,000 unit batch) of this wireless sensor system make it particularly attractive for phonocardiography and other sensing applications. The experimental results of sensor signal analysis using several signal characterization techniques suggest that this wireless sensor system can capture both fundamental heart sounds (S1 and S2), and is also capable of capturing abnormal heart sounds (S3 and S4) and heart murmurs without aliasing. The results of a denoising application using Wavelet Transform show that the undesirable noises of sensor signals in the surrounding environment can be reduced dramatically. The exercising experiment results also show that this proposed wireless PCG system can capture heart sounds over different heart conditions simulated by varying heart rates of six subjects over a range of 60–180 Hz through exercise testing.

## 1. Introduction

**Figure 1.**
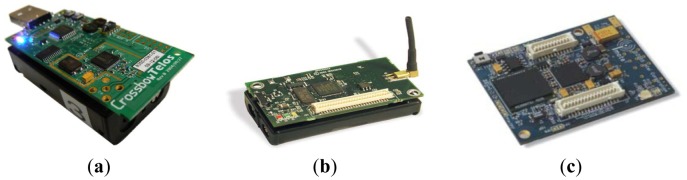
Sensor Networking Platforms. (**a**) Telos; (**b**) MicaZ; (**c**) Imote2.

The emerging Wireless Sensor Network (WSN) technologies have begun to advance the monitoring and control of many complex, real-world systems, such as in structural and mechanical [1,2], environmental [3–5], healthcare [6–8], and military applications [9]. A WSN consists of multiple small, foot-print wireless devices called “sensor nodes,” each of which is typically composed of a radio (RF) transceiver, microcontroller, memory unit, and battery. WSN technologies using Zigbee protocol (IEEE 802.15.4) allow sensor nodes to collect data by using low-cost microcontrollers and Radio Frequency (RF) transceivers. Some of the lightweight Zigbee WSN platforms include Mica2 [10], MicaZ [11], TelosB [12] for low-end, and Yale's XYZ [13] and Intel's IMote2 [14] for high performance applications (see Figure 1).

Pertinently, the WSN technologies offer significant potential to transform healthcare monitoring practice [15–19]. WSN allows medical professionals to remotely track and monitor a patient's physiological signals, such as blood pressure, heart rate, ECG, and heart sound, continuously over an extended period of time. Especially in the case of critical-care patients who require a round-the-clock monitoring system, WSN devices allow medical doctors to detect abnormal signals in a timely manner. WSN devices also enable patients to have greater freedom of movement and less discomfiture compared to traditional wired devices. Additionally, WSN devices may ultimately be used by patients for self-diagnosis.

According to the British Medical Bulletin [20], cardiovascular diseases are the leading cause of death globally. Significant efforts have been made to address the diagnostics of various cardiovascular disorders using a variety of sensors, including electrocardiography (ECG), magnetic resonance imaging (MRI), and phonocardiography (PCG). In particular, PCG is a common method for a physician or medical doctor to analyze a patient's heart. PCG techniques use heart sound signals collected from a highly sensitive microphone for heart condition monitoring. A PCG sensor [21,22] offers certain advantages over other physiological sensors, including ECG and MRI, because acoustic monitoring of a heart condition using PCG is harmless and nonintrusive, the setup is lightweight, and a relatively low level of experience and skill is needed to set up the system and acquire the signals. The PCG recording also requires only a single probe and does not use wires, the time required to set up PCG recording is shorter, compared to ECG and MRI. More importantly, PCG offers the ability to quantitate the sounds made by the heart providing information not readily available from more sophisticated tests. The ECG, which reveals the electrical activity of the heart, is used to detect heart abnormalities by drawing a graph of the electrical impulses moving through the heart. Although acquisition of ECG is noninvasive and painless, and the ECG signals provide useful information of the electrical activity of the heart, these signals do not always permit an accurate diagnosis due to multiple factors affecting electrical activity of the heart. This method also requires relatively high level of experience and skill to set up the system and perform an analysis. Hence, ECG and MRI are not normally used unless a problem has been previously detected by PCG or auscultation [23]. Although, a PCG sensor offers certain advantages over other physiological sensors, heart sounds collected using PCG usually include undesirable noises from other parts of the body and also from the surrounding environment. To address this issue, many techniques have been developed to remove undesirable noises from heart sounds collected using PCG [24,25].

Some of these factors and benefits have led to the development of wireless PCG sensor systems [26] to facilitate remote diagnostics by medical professionals and self-diagnosis by patients themselves. A wireless PCG sensor is highly suitable for high-risk populations, including those in critical care and those subjected to intense physical activity, as in sports training. Many cardiovascular diseases are treatable in their early stages but can develop rapidly from their inception to become highly life threatening. Early detection of these diseases may be necessary to facilitate cost-effective treatment and to improve the chances of complete recovery. Many classes of WSNs can help with the continuous PCG data acquisition necessary to facilitate such early detection of disorders.

Some of the recent developments in sensor and wireless communication techniques for PCG applications are the Bluetooth-based wireless data acquisition system for Phonocardiogram [27] and wireless medical stethoscopes [28,29]. These devices are developed for use with Bluetooth wireless technology. The advantage of using the Bluetooth protocol is that it allows a very high data rate compared to other protocols such as Zigbee and Wi-Fi. However, it requires high-performance microprocessors, which consumes high energy, have a shorter battery life, and tend to be somewhat expensive compared to Zigbee devices. The objective of this research is to develop an affordable wireless PCG sensor system that is able to capture PCG signals from human heart, and can be used for continuous monitoring and risk assessment of subjects at risk, including diseased populations and those subjected to extreme physical activity levels, as during athletic training sessions, without curtailing their mobility.

In order to develop this wireless PCG sensor system, several commercially available WSN platforms, such as Tmote, TelosB, MicaZ, and Imote2, were considered as candidates for the wireless module component of a system. However, these platforms are disadvantaged by one or both of the following: (i) high costs; and (ii) their inability to support the required sensory data streaming bandwidth. In this paper, a wireless sensor system based on the Zigbee protocol is presented. This wireless sensor system has been designed and developed in-house using off-the-shelf components and open source software for remote and mobile applications [30]. The small form factor (3.75 cm × 5 cm × 1 cm), high throughput, and low cost of this wireless sensor system make it particularly attractive for phonocardiography and other sensing applications. The results of sensor signal analysis using several signal characterization techniques suggest that the wireless sensor system can capture both fundamental heart sounds (S1 and S2), and is also capable of capturing abnormal heart sounds (S3 and S4) and heart murmurs without aliasing.

The organization of the remainder of this paper is as follows: In Section 2, the overview of WSN platforms and phonocardiography sensors is presented. The proposed wireless platform architecture and sensor integration are described in Section 3. In Section 4, strategies for improving the sensor's data streaming rate are provided. The PCG sensor implementation details and validation studies are shown in Section 5. The summary of this paper is provided in Section 6.

## 2. Overview of WSN Platforms and Phonocardiography Sensors

The WSN platforms can be categorized into three main groups, namely, Advanced RISC Machine (ARM)-based platforms, microcontroller-based platforms and RF integrated platforms [31]. ARM-based platforms are designed mainly for applications that require considerable processing power. For instance, Intel Imote2 uses an Intel PXA271 processor which is an ARM-based processor. This processor can provide a very high sampling data rate and is aimed at applications involving high-performance computations, where high bandwidth is required. However, compared to other platforms, this type of WSN platform also consumes high energy. In the case of applications where power consumption is a major consideration, microcontroller-based platforms are considered much more suitable. The MicaZ platform is specifically optimized for low-power, battery-operated networks. This platform uses an ATmega processor, which is a relatively slow processor but requires much less power compared to ARM processors. RF integrated platforms are designed for applications where a small form factor is required. However, the disadvantage of this type of platform is that the processor is relatively slow compared to other platform types.

**Figure 2.**
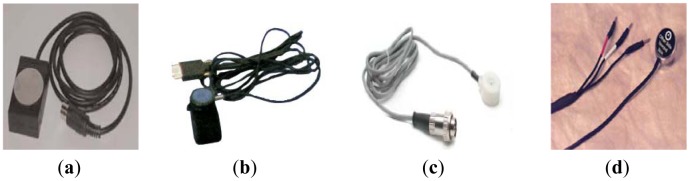
Example of phonocardiography sensors (**a**) HSM-300 Heart sounds Monitor from IWORK; (**b**) Heart sounds microphone model 50-4724 from MindWare; (**c**) MLT 201 Cardio Microphone from ADinstruments; (**d**) Physiological Sounds Microphone Model AH153 from Biopac Systems.

A wireless sensor system for PCG applications consists of two main elements: a wireless platform and a PCG sensor. The most common PCG sensor for medical applications is a stethoscope. A physician places a stethoscope on the surface of the patient's body to listen to heart sounds. Other types of phonocardiography sensors, including heart sound and cardio microphones, have also been used in cardiological research as shown in Figure 2.

**Figure 3.**
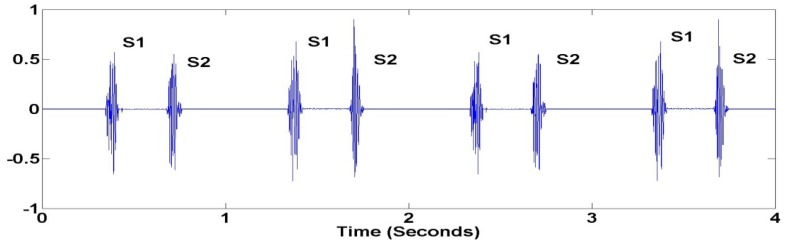
Fundamental Heart Sounds (S1 and S2).

The purpose of these sensors is to detect heart sound signals from the human body. The heart sound data is independently sampled using a microprocessor on a WSN platform at an appropriate sampling rate to detect significant characteristics of the heart signal. Generally, heart sounds lie in the frequency range between 20 Hz and 2 kHz. During heart auscultation, four types of heart sounds can possibly be heard. The most fundamental heart sounds are the first and second heart sounds (S1 and S2) as shown in Figure 3.

**Figure 4.**
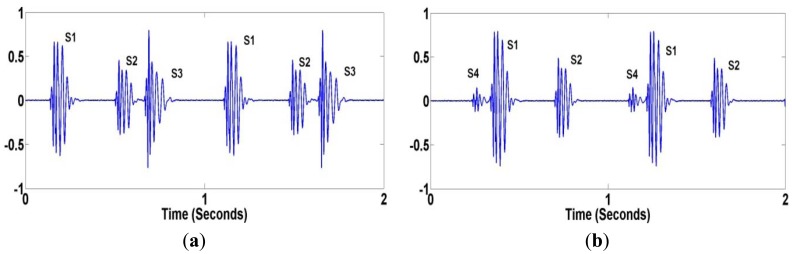
Abnormal Heart Sounds (**a**) S3; (**b**) S4.

The abnormal heart sounds (S3 and S4) shown in Figure 4(a,b) are the sign of cardiovascular disorders, but also can sometimes be heard in healthy children and young adults. The frequency content of heart sounds lies in the range of 20–200 Hz for S1 and S2, and less than 50 Hz for S3 and S4. Also, an extra or unusual sound, called a heart murmur, can be heard during a heartbeat. A heart murmur frequency lies anywhere between 20 Hz and 2 kHz.

## 3. Wireless Platform Architecture and Phonocadiology Sensor Integration

**Figure 5.**
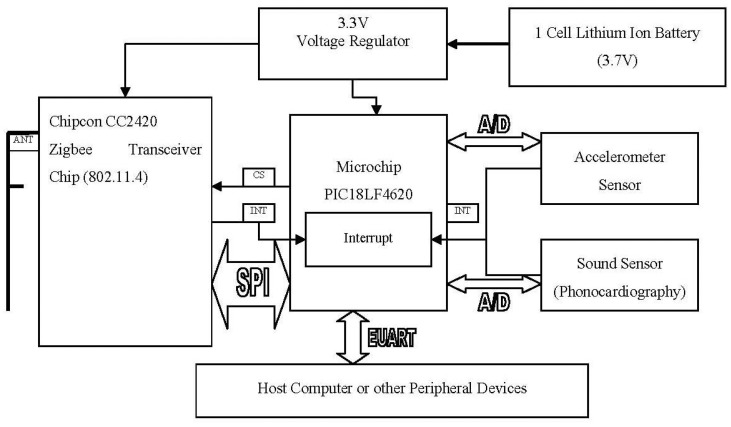
Architecture of Wireless Sensor Platform.

As the objective of this research is to develop an affordable wireless PCG system, we decided to develop an in-house wireless platform for phonocardiography applications. The proposed platform is based on the “PICDEM' developer kit from Microchip [30]. The main components of the proposed platform consist of the Microchip processor PIC18F4620 and a Chipcon Zigbee CC2420 wireless transceiver radio. As shown in Figure 5, the two boards, the processor and wireless transceiver, are sandwiched together providing a complete platform with the radio unit communicating with the processor through an SPI bus.

The core of this wireless platform is the PIC18F4620 microprocessor. This processor incorporates a number of features making it attractive for embedded wireless sensor network operations. Some of the features include nanoWatt technology for low power operations, multiple oscillator options for operating at different clock speeds, advanced memory management for increased programming flexibility, enhanced addressable USART, a 10-Bit A/D converter, and an extended watchdog timer. The processor's maximum clock speed is 40 MHz and it is capable of sustaining 8 MIPS with a supply voltage of 5 V.

The processor has three programmable interrupt inputs, which allows us to monitor three different signals simultaneously. Currently, we use one of the interrupts for invoking the processor from sleep. The application software decides when to put the processor to sleep during which nanoWatt technology is invoked. As the processor is sleeping, the interrupt pin is the only active pin which can wake the processor up once it detects changes in the electrical signals on the pin. The ability to place the processor on standby is critical for conserving battery power. The processor provides 13 channels for 10-bit analog to digital conversion (ADC). All of the A/D pins can be programmed as both input and output pins. Three pins can be programmed for multiplex with an alternate function from the peripheral features on the device. In addition, an Enhanced Universal Synchronous Asynchronous Receiver Transmitter (EUSART) module is available in this processor. The EUSART provides more robust, reliable, and faster data transfers between two devices when compared to traditional UAR. The EUSART can be programmed either as a full-duplex asynchronous or a half-duplex synchronous system. This flexibility is quite advantageous because most A/D or D/A integrated circuits as well as most serial EEPROMs communicate using a half-duplex synchronous method.

The PIC18LF4620 provides seven operating power modes and hence may support more efficient power management. The maximum sink/source current rating of the processor is 24 mA, which is quite useful when considering the fact that most sensors in the market today (vibration, light, sound, heart, *etc.*) require a current source less than 24 mA. Another interesting feature of the processor is that it supports direct memory addressing. Direct addressing allows the user to access any location in the processor's main memory without a fixed address in the instructions. This access is achieved by using the File Select Registers (FSR) as pointers to the desired memory location. The FSR is located in the processor RAM as a special function register. It can be manipulated by a user program, which makes this feature quite useful in implementing data structures such as look-up tables and arrays.

**Figure 6.**
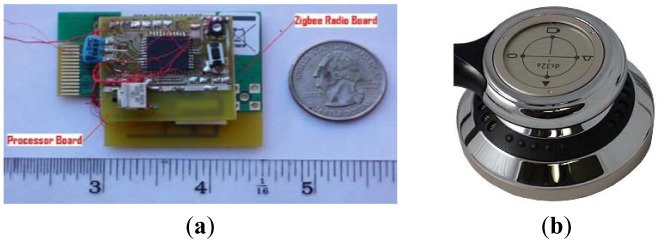
(**a**) The Actual Wireless Platform; (**b**) Electronic Stethoscope Model ds32a.

Several types of sensors have been tested for a wireless PCG sensor system [32]. The diaphragm of electronic stethoscope model ds32a from Thinklabs [33] was selected as the best option for the PCG sensor because of its noise cancellation technology and capability to detect acoustic sounds at both low and high frequencies of the heart. Figure 6 shows the actual wireless platform and the diaphragm of the stethoscope used for the wireless PCG sensor system.

With this explanation of the wireless sensor platform based on the PIC18LF4620 and CC2420 chips, the following subsections are devoted to describing several aspects of the default performance of this proposed wireless platform, including form factor, cost estimate, power consumption, and sensory data streaming rate.

### 3.1. Form Factor

As mentioned earlier, the PIC18LF4620 processor is attached to the Chipcon CC2420 radio transceiver board. The transceiver board is connected to an inverted F antenna designed around a printed circuit board. The radiation pattern of this antenna is similar to that of a monopole antenna with a ground plane. The efficiency of our inverted F antenna is about 0.6 and the normal input impedance is approximately 50 Ω. The antenna efficiency was computed by the ratio between radiation resistance and total resistance of the inverted F antenna, *E* = *R_radiation_*/*R_total_*, where *E* is the antenna efficiency, *R_radiation_* is radiation resistance and *R_total_* is total resistance of the inverted F antenna. As shown in Figure 6(a), the entire unit is about 3.75 cm wide, 5 cm long, and 1 cm thick.

### 3.2. Cost Estimate

The main components in the wireless sensor platform are the PIC18LF4620 microprocessor from Microchip and the Chipcon CC2420 Zigbee radio transceiver chip. The printed circuit boards (PCBs) used in the fabrication are two sided, and the overall circuit employs a minimal number of auxiliary components (in terms of resistors, capacitors and inductors). Consequently, the PCBs do not require multiple layer traces. When the unit is manufactured in large quantities (1,000 or more pieces), it is estimated that the cost per unit can be $13 or less. About 70% of the unit cost is the combined cost of the microprocessor ($5.15 for 1,000 units) and the Zigbee transceiver chip ($4.12 for 1,000 units). The rest of the cost is for the supporting components, including the PCG sensor, PCB, capacitors, resistors, inductors, and assembly.

### 3.3. Power Consumption

The power consumed by the wireless sensor platform is measured based on the low side current sensing technique. This technique has been widely acknowledged to be precise, straightforward, and easy to implement. Two parallel 1 Ω resisters (for an equivalent resistance of 0.5 Ω) were placed between the load and the system ground. This equivalent resister acts as a sense resister. The voltage drop across this resister is measured and Ohm's law is used to calculate the current passing through the resister, which is same as the current consumed by the wireless sensor platform. A high precision Fluke digital volt meter and oscilloscope were used to measure the voltage drop across the resister and 2 AA batteries were used as the power source. The final measured current consumption of the sensor platform equipped with a 4 oscillator is given in Table 1.

### 3.4. Sensory Data Streaming Rate

To develop an effective wireless sensor system, it is important that the developed sensor platform possess the ability to transmit heart sound samples at rates at least as high as 4,000 Hz. Our initial tests of the capabilities of the proposed sensor platform to transmit data samples at a high rate showed that the default application throughput was 3.73 kbps at a sampling rate of 233 Hz, with each sample being 10 bits wide.

From the default performance described above, we have shown that the proposed sensor platform has acceptable form factor and cost estimates for being employed in a wireless phonocardiography system. The power consumption of the proposed platform is between that of Tmote/TelosB/MicaZ and Imote platforms. However, the issue of sensor data quality remains to be resolved. In order to achieve our goal in terms of sampling rate, we focused on carrying out various refinements to the software running the sensor platform as described in the following section.

## 4. Strategies for Improving the Sensor's Data Streaming Rate

As shown in Figure 5, a 10 bit ADC can receive analog sensory input from a phonocardiography (heart sound) sensor. The idea is that the ADC's output is fed to the microcontroller, which passes it on to the Zigbee protocol stack for transmission. The ADC is very fast and is capable of sampling the sensory data at a rate of 100 kHz. This rate, in conjunction with the default streaming rate, implies that while the Zigbee stack, running on the microprocessor, can receive samples at a rate of 100 kHz, it can push these samples to the transceiver only at a rate of 233 Hz. Therefore, the performance bottle neck, in terms of meeting the required sampling rate, lies with the Zigbee protocol stack.

Having identified the possible bottleneck, we present the following strategies for improving the performance. It is to be noted that each of the five strategies is rather independent of the others and hence they can be applied in unison to achieve maximum performance gains.

### 4.1. Increasing the Packet Size during Transmission

Sensory samples from the ADC are sequentially packed into the payload field of the Zigbee packets. When the payload reaches a certain size, the packet is transmitted. With each ADC sample occupying two bytes, a maximum of *P/2* samples can be packed in a packet with a pay load width of *P* bytes. In applications requiring high sampling rates, the time interval between two consecutive sensor samples is considerably lower than the time required to transmit a packet. Therefore, if the packet size is increased, more samples can be packed in each packet. This reduces the time spent in packet transmissions, which in turn can increase the overall sampling rate.

Of course, the time taken by the Zigbee protocol stack to push a packet to the transceiver can increase with the packet size, which may undermine the sampling rate gains achieved by the packet size increase. However, our studies showed that, while it takes a longer time to transmit a bigger packet, the gains achieved in terms of sampling rate are considerable, making it worthwhile to increase the packet size. While the maximum packet size in the Zigbee can be as high as 127 bytes (including the packet header), we found that increasing the packet's payload beyond 96 bytes makes the microcontroller unstable. A possible reason for this instability could be the size of the buffers and registers in the Zigbee transceiver system. Consequently, we fix the maximum packet payload size as 96 bytes.

### 4.2. Zigbee Stack Refinements

The Zigbee protocol standard was garnered towards applications with a low sampling rate and low energy consumption. The phonocardiography application demands a high sampling rate. The obvious mismatch in the sampling characteristics requires that the Zigbee stack may have to be refined to meet the requirements of the application at hand. Some of the features in the default Zigbee stack are unnecessary in the phonocardiography application. Specifically, the sensory data flow is predominantly unidirectional—*i.e.*, from the sensor node to a base station. There may not be much data flow in the reverse direction. However, by default, the Zigbee assumes a bi-directional data flow between two communicating devices. Consequently, data synchronization primitives are executed by the Zigbee stack by default. This implies that whenever a node attempts to transmit sound data to the base station, the data synchronization primitives necessitate additional message exchanges between the two, consuming additional power and, more importantly, time. In phonocardiography applications, for reasons described earlier, such unnecessary data synchronization primitives can be safely removed without undermining the protocol operation, thereby improving the protocol turnaround time.

### 4.3. Employing Data Compression

As explained above, each sensory data sample occupies two bytes in the 96 byte long payload of packets transmitted by the sensor node. If by some means, the real estate for each sample can be lowered, then more samples can be packed into a single packet, thereby increasing the overall sampling rate from the application's perspective. In order to achieve this, we resort to data compression. While it is tempting to apply sophisticated data compression techniques [34], we adopt a rather simple compression technique as follows.

**Figure 7.**
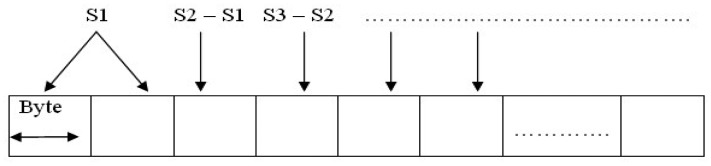
Packaging Data Samples with Compression.

As shown in Figure 7, the first sample (say *S*_1_) to be loaded in each packet is always given the original real estate of two bytes. The second sample (*S*_2_) is loaded into the packet not in its original form, but in a differential form: *i.e.*, in byte 3 of the packet, information pertaining to *S*_2_ is loaded as *S*_2_−*S*_1_; sample *S*_3_ is loaded into byte 4 of the packet as *S*_3_−*S*_2_; and so on. The lowest seven bits of byte *i*, 1 < *i* ≤ 96 in the packet, are used to represent the magnitude of the difference *S_i_*_−1_–*S_i_*_−2_, while the highest bit is used to indicate the direction of the difference (positive or negative). It is easy to see that, when the macro tag receives the first sample and the subsequent differences, it can easily reconstruct all the samples.

Such a scheme can work quite well in scenarios where the difference between two consecutive sensory samples *S_i_* and *S_i_*_+1_ (that are 10 μs apart) is no more than 70% of *S_i_*. Such a compression technique can certainly be extended across packets to reduce the two byte real estate for the first sample to one byte. However, since the transmission medium is wireless, if a single packet is lost on account of transmission/channel errors, decoding subsequent packets will become difficult owing to the dependency on the lost packet. Therefore, we restrict the sample dependency to be within a packet by allocating two bytes for the first sample. Using this compression technique, it is possible to pack 95 sensory data samples in a single packet, as opposed to 48 samples without compression.

### 4.4. “Store-and-Transmit” Strategy

The ADC built in to the processor is capable of sampling at rates as high as 100 kHz; however, the Zigbee stack running on the processor is slow in transmitting the sampled data. Therefore, if an application does not require continuous sensory inputs and can tolerate data delivery latencies of up to a few seconds, a “store-and-transmit” strategy can be devised to achieve high sampling rates. The ADC can be allowed to sample the sensory input at the maximum possible rate, and the sampled data can be stored in a buffer. The sampling can be temporarily suspended when the buffer is full, and the Zigbee stack can be invoked to transmit the data stored in the buffer. The sampling can resume when the buffer is emptied. Such a buffer and transmit strategy can work providing there is enough buffer space to satisfy the application's requirements.

Our studies indicate that the data memory of the PIC18F4620 processor is 4K bytes, divided into 16 banks. Therefore, by default, the Zigbee stack cannot access a buffer larger than 256 bytes due to restrictions placed by the bank boundaries. Nevertheless, by modifying the linker script and using preprocessors, it is possible to combine/merge several banks into a larger memory block. However, we discovered that very few of the 16 memory banks are free and can actually be consolidated into a single large memory block. Of the 16 memory banks available, banks 1 through 8 are reserved for the processor firmware's heap. Bank 9 is reserved for the stack, while bank 10 is reserved for the Zigbee stack's receiver buffer. The remaining banks–bank 0, banks 11–15 are free for consolidation. Thus the actual in-situ memory available for consolidation in the PIC18F4620 processor is too limited to apply the buffer and transmit strategy.

While one can resort to connecting additional memory chips to the sensory unit to overcome this shortage, external RAMs will consume additional real estate and power. Consequently, we do not adopt such an approach. Therefore, the “store-and-transmit” strategy is not employed in our experiments.

### 4.5. Increasing the Processor Clock Speed

In order to reduce the overall execution time of the Zigbee stack (and thus decrease the packet turnaround time), we resort to increasing the clock speed of the microcontroller. The default CPU clock for PIC18F4620 is 16 MHz, fed from a 4 MHz oscillator crystal. The data sheet for PIC18F4620 indicated that the microcontroller's CPU can function at rates as high as 40 MHz. In order to realize the complete benefits of the clock frequency increase, we used a 10 MHz oscillator and a CPU clock rate of 40 MHz. The results were very encouraging and can be seen in Table 2. It is to be noted that all the results given in the table employ strategies in Sections 4.1 and 4.2. The results of adopting strategies in Sections 4.3 and 4.5 are also indicated in Table 2.

A possible drawback of increasing the processor clock speed is that the power consumption will increase, as can be seen from Table 3. While the power consumption of the proposed sensor platform is higher than that of other sensor platforms, we believe that the lower cost of the unit partially offsets this increase by allowing additional units to be deployed, thereby achieving a lifetime equivalent to that which may achieved by commercial units.

To present the robustness of the sensor performance of the proposed sensor system, Table 4 shows a comparison of the sensor performance of the proposed sensor system and several existing sensor platforms. As seen in Table 4, the proposed sensor system can significantly improve data streaming rate (exceeds 6,000 Hz) while reduces the cost ($13 per unit) effectively. Another advantage of the proposed sensor system is that it runs a nonproprietary firmware which allows a high programming flexibility. Most of the commercial wireless sensor platforms, including TelosB, T-mote and MicaZ, support TinyOS [35] open source hardware. These WSN platforms can be programmed with binary codes obtained from TinyOS. However, these WSN platforms run a proprietary firmware (the boot software) due to which it is not possible to freely use these platforms. Only possible drawback of this proposed sensor system is high power consumption as stated previously. However, the proposed sensor system can also be adjusted to achieve low power consumption by reducing CPU clock speed for applications requiring lower sampling frequency.

## 5. PCG Sensor Implementation Details and Validation Studies

The proposed wireless platform described in previous sections was used to collect heart sounds using the diaphragm of a stethoscope as the PCG sensor. The details of the signal acquisition and conditioning, as well as experimental performance evaluation are provided in this section.

### 5.1. Heart Sound Signal Analysis

**Figure 8.**
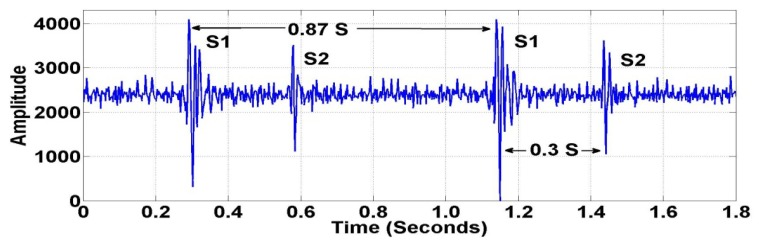
Location, Time Interval of Fundamental Heart Sounds (S1 and S2).

**Figure 9.**
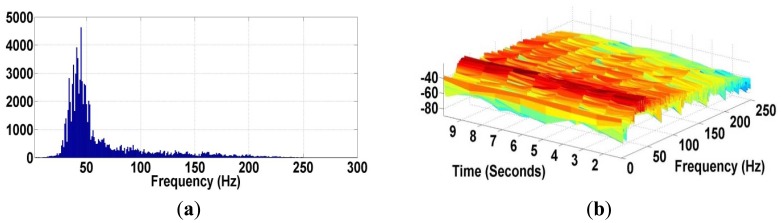
(**a**) Frequency Content of Wireless Sensor Signal using FFT; (**b**) Segmentation of Wireless Sensor Signal using STFT.

Generally, the characteristics of heart sounds include S1 and S2 locations, the number of components for each sound, their frequency content, and their time interval. As shown in Figure 8, the location, time interval, and number of components can be validated easily using the local peaks (extremas) of the sensor signal. In order to measure the quality of the wireless PCG signal, several signal characterization techniques were used to test the frequency characteristics of the sensor signals, including the Fast Fourier Transform (FFT) [36] and the Short-time Fourier Transform (STFT) [37]. The FFT is used to convert the original sensor signal from the time domain to the frequency domain. It is an efficient algorithm to compute the Discrete-Time Fourier Transform (DTFT) and its inverse. The mathematical definition of the DTFT of time series *x*(*t*), where *t* = 1,…,*N*, is given in Equation (1):

(1) $$X(\omega )=\sum_{t=1}^{N}x(t)e^{-i\omega t}$$

where *t* is time and *ω* is frequency component, respectively. The DTFT of *x*(*t*) provides an approximation of the Continuous-Time Fourier Transform of time domain signal *x*(*t*). One disadvantage of the Fourier Transform is that it only determines the frequency components of a signal. However, it can’t tell us about their location in time. So, it is not adequate to the heart sound which presents non-stationary characteristics. Figure 9(a) shows the frequency contents of the wireless sensor signal using the FFT.

The analysis of non-stationary signal requires time dependency analysis. The STFT is used to map the signal in time and frequency domain together. The STFT transforms the sensor signal using the Fourier transform as the window slides along the time axis. The mathematical definition of the STFT can be written as:

(2) $$X(\tau ,\omega )=\int_{-\infty }^{\infty }x(t)w(t-\tau )e^{-j\omega t}dt$$

where *w*(*t*) is the window function and *x*(*t*) is signal in time domain. *X*(*τ*,*ω*) is basically the FT of *x*(*t*)*w*(*t* − *τ*). A complex function represents the phase and magnitude of the signal over time and frequency.

The frequency contents of the wireless sensor signal vary from around 20 Hz up to 200 Hz, corresponding to the frequency range of the fundamental heart sounds (S1 and S2). The STFT technique in Figure 9(b) indicates the segmentation of heart sounds via the intensity or color of points in the image. The frequency character of the wireless sensor signal also indicates the amplitude of particular frequency at a particular time. The more intense color area in the STFT pot corresponds to the first heart sound (S1), which has a frequency peak around 35 to 60 Hz.

Although the wireless phonocardiography sensor system is capable of detecting both fundamental heart sounds (S1 and S2) without aliasing, heart sounds collected from the human body usually include undesirable noises from other parts of the body and also from the surrounding environment. Significant efforts [38–40] have been made to address this issue using denoising techniques. In this paper, a Wavelet Transform [41] is used via a thresholding operation to remove undesirable noises from collected heart sounds. The process of this noise reduction technique is to produce a finite number of wavelet coefficients using a discrete wavelet transform (DWT), apply a thresholding operation which removes some coefficients of the DWT, and then use the inverse discrete wavelet transform (IDWT) to reconstruct the signal. Basically, an algorithm of this procedure is as following:

1. Compute the first two sets of coefficient using low- and high-pass filters. The length of both low- and high-pass filters is equal to 2*N*, where *N* is the length of heart signal.
2. The approximation and detail coefficients are computed using the convolution of heart signal with both filters. Then, the next step is to decompose approximation coefficient of the first level to two set of coefficients (approximation and details) in the second level. The process continues iteratively until reaching the defined level.
3. Applying thresholding operation by specifying the threshold for each coefficient of wavelet and then use the inverse discrete wavelet transform (IDWT) to reconstruct the signal.

**Figure 10.**
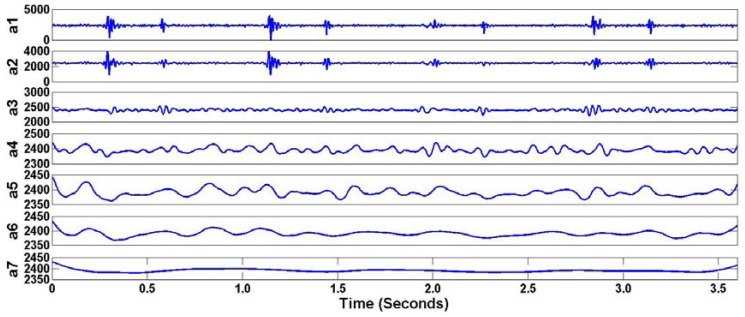
Seven Approximation Coefficients (a1–a7) of Heart Sounds using Daubechies Wavelet.

**Figure 11.**
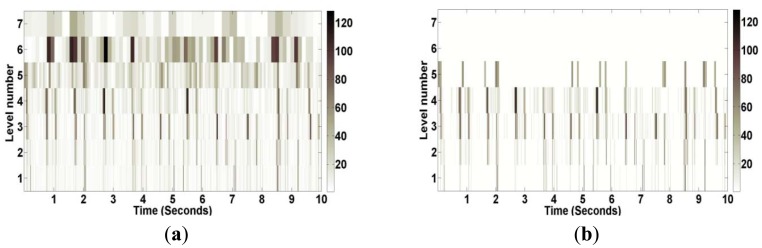
(**a**) Original and (**b**) Thresholded Coefficients of Heart Sounds using Daubechies Wavelet.

**Figure 12.**
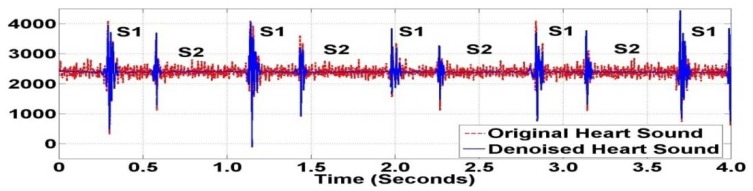
Original and Denoised Heart Sounds using Daubechies Wavelet.

Many types of wavelets and levels of decomposition can be selected for the thresholding operation. From the experimental performance, order five of Daubechies family at level seven was selected for decomposition. During the decomposition process, seven approximation coefficients of Daubechies wavelet can be obtained as shown in Figure 10. Original and thresholded coefficients of heart sounds using wavelet denoising techniques are shown in Figure 11. This application can be considered a preprocessing application of heart sound data analysis. Figure 12 shows the original heart sound collected from a wireless PCG sensor system and the denoised heart sound using the thresholding operation of wavelet transform. The results show that undesirable noises from the surrounding environment are reduced dramatically. The denoising application using the wavelet thresholding operation is very useful for heart sound analysis by auscultation. However, one problem may arise due to the difficulty of noise reduction for heart sounds. Unlike the fundamental heart sounds (S1 and S2), the abnormal heart sounds (S3 and S4) are low-frequency and low-energy signals that can be buried under the noise from surrounding environment. In order to remove undesirable noises from abnormal heart sounds, additional references, such as timing and frequency information, may be required for undesirable noise removal using wavelet thresholding operation.

### 5.2. Experimental Studies of the Wireless PCG Sensor in Exercise Testing

The common procedure for listening to the heart is for auscultation to proceed in a logical manner over four general areas on the anterior chest, beginning with the subject in a supine position: the aortic region, pulmonic region, tricuspid region, and mitral region. Rate and rhythm, value functioning, and anatomical defects are the common pieces of information which can be gathered from listening to the heart. The main area of this experiment was the pulmonic region because this area provides the clearest fundamental heart sounds (S1 and S2), compared to other regions. As discussed in the introduction section, this wireless PCG sensor system can lead to the development of remote- and self-diagnosis systems for high risk populations, including those in critical care and those subjected to intense physical activity, as in sports training.

The experiments were conducted in two phases. Phase 1 experiments aim to test the performance of the wireless sensor platform and the quality of the heart sound signal for data analysis. Six subjects were collected the heart sounds using both wired and wireless devices simultaneously. The subject was placed in a supine position, and the wireless PCG sensor was placed on the chest in the pulmonic region. The heart sound was recorded for 10 s with the breath stopped during measurement. For the preliminary measurement, the wireless heart sound signal was acoustically amplified by the PCG sensor and then sampled independently using the ADC and microprocessor of the proposed wireless platform. The signals from these experiments were used to test the performance of the denoising procedure.

In Phase 2, we experimented with the use of a proposed wireless PCG sensor system, as in a sports training application, with six subjects. Each subject was assigned to run on the treadmill at a speed for 7 m/h for 10 min. The heart sound signals were collected before and after the training experiment. The procedure for this application is the following:

- Collect heart sounds from subjects using the auscultation procedure under normal conditions for 30 s (use the wireless PCG sensor system and wired PCG sensor (stethoscope) to collect heart sounds in the pulmonic region as subjects are in a supine position).
- Redo the measurement after subjects complete running on a treadmill for 10 min (same speed, inclination, and all setup) when the signal is known to be highly nonstationary.
- Continue measuring the heart sounds until the heart rate goes to the normal rate (before running) for 10 min.

**Figure 13.**
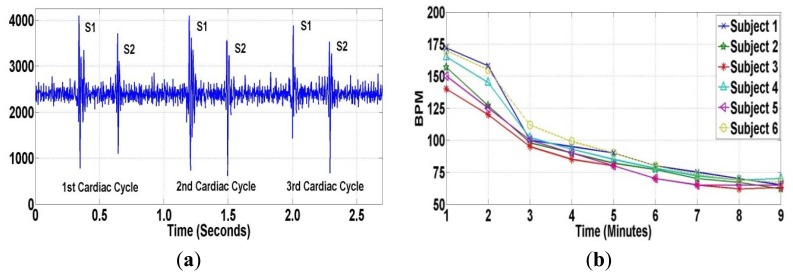
(**a**) Heart Sounds before Experiments; (**b**) Heart Rate (BPM) after Experiments.

Many studies [42–44] have reported that the heart rate is one of the risk factors in cardiovascular disease. An elevated heart rate has been found to be a powerful predictor of death in patients with coronary artery disease (CAD), myocardial infarction, and congestive heart failure. Collecting heart sounds before and after training experiments provides useful information for analyzing the human heart. Fundamental heart sounds (S1 and S2) can be located using local peaks and timing as shown in Figure 13(a). Figure 13(b) shows the heart rate of all subjects after the training experiment. From the experimental results, the proposed wireless PCG sensor system, compared to a wired PCG sensor, can capture heart sounds data without data loss.

## 6. Summary and Conclusions

This paper presents the design and testing of a wireless sensor system developed using a Microchip PICDEM developer kit to acquire and monitor human heart sounds for phonocardiography applications. The system can serve as a cost-effective option to recent developments in wireless phonocardiography sensors that have primarily focused on Bluetooth technology. This wireless sensor system has been designed and developed in-house using off-the-shelf components and open source software for remote and mobile applications. The small form factor, high throughput, and low cost of this wireless sensor system make it highly suitable for several applications, including phonocardiography applications. In this paper, we present an integration of this wireless sensor system and a phonocardiography sensor providing a wireless heart sound data acquisition system for PCG applications.

In order to achieve high data throughput using this wireless sensor platform, five strategies for improving the sensor's data streaming rate have been proposed: (i) increasing the packet size during transmission; (ii) Zigbee stack refinements; (iii) employing data compression; (iv) a store-and-transmit strategy and (v) increasing the processor clock speed. Laboratory tests indicate that the proposed wireless sensor system can generate relatively high throughput which is capable of sampling and transmitting sensory data at frequencies more than 6,000 Hz. The experimental results for the phonocardiography application show that the wireless sensor system is capable of detecting both fundamental heart sounds (S1 and S2) without aliasing. Heart sound analysis using various techniques is also presented in this paper, including the Fast Fourier Transform (FFT), Short-Time Fourier Transform (STFT) and Wavelet Transform (WT). The use of a Wavelet Transform domain filtering technique via a thresholding operation shows that undesirable noises from the surrounding environment are reduced dramatically. The denoising application using a wavelet thresholding operation is very useful for heart sound analysis by auscultation.

Finally, this paper also presented the use of a wireless sensor system for sports training applications. The experimental results indicate that this wireless sensor system can lead to the development of remote diagnostics and self-diagnosis systems for monitoring heart sounds in high risk populations, including those in critical care and those subjected to intense physical activity, as in sports training.

## Figures and Tables

**Table 1.** Measured Current Consumption.

**Processor**	**Radio**	**Current Drawn (mA)**

ON	ON	39
ON	OFF	27
OFF	ON	25
OFF	OFF	∼0

**Table 2.** Sensory Data Sampling Rate.

**Oscillator Speed**	**CPU Clock Speed**	**Compression**	**Application Sampling Rate (Hz)**

10 MHz	40 MHz	Yes	6,520
10 MHz	40 MHz	No	2,597
8 MHz	32 MHz	Yes	3,135
8 MHz	32 MHz	No	1,665

**Table 3.** Power Consumption.

**Crystal Clock Rate**	**CPU Clock Rate**	**Power Consumption (mA)**

10 MHz	40 MHz	51
8 MHz	32 MHz	39

**Table 4.** Comparison of alternative WSN Platforms.

**WSN Platforms**	**TelosB**	**T-Mote**	**MicaZ**	**Proposed Sensor System**

CPU Type	MSP430	MSP430	Atmel	PIC18F4620
CPU Clock	8 MHz	8 MHz	16 MHz	40 MHz
Radio Chip	CC2420	CC2420	CC2420	CC2420
Data Streaming Rate	<1,000 Hz	<1,000 Hz	<1,000 Hz	6520 Hz
Power Consumption	24.8 mA	23.0 mA	27.7 mA	51 mA
Programming Flexibility	Moderate	Moderate	Moderate	High
Size w/o batteries (cm) W × L × T	3 × 7 × 0.5	3 × 7 × 0.5	3 × 6 × 0.5	3.75 × 5 × 1
Expandability	Yes	Yes	Yes	Yes
Firmware	Proprietary	Proprietary	Proprietary	Nonproprietary
Cost	$99	$89	$99	$13
